# Principles of Medicine

**Published:** 1857-11

**Authors:** 


ART. VI.—*Principles of Medicine. An Elementary View of the Causes, Nature, Treatment, Diagnosis, and Prognosis of Disease. With brief remarks on Hygienics, or the Preservation of Health.* By CHARLES J. B. WILLIAMS, M.D., F.R.S. A new American, from the Third and Revised London Edition. 8vo. pp. 486. Philadelphia: Blanchard & Lea. 1857.

No one can peruse the work of Dr. Williams without being convinced that it is a most masterly production. Written in a style remarkable for simplicity and clearness, every page is replete with profound thought, solid matter, and sound judgment. It is eminently a work of principles, a knowledge of which we wish we could infuse into the minds of the profession, as heat and light are diffused through air and water, imparting to them their animating, life-stirring, invigorating influence. It is just such a book as every preceptor should put into the hands of his pupils, as a guide to the study of medicine and surgery; it is, in our judgment, decidedly the best treatise on pathology and practice that has been written in modern times.

That the work of Dr. Williams is appreciated in this country is sufficiently evidenced by the fact, that it has already passed through a number of reprints; but that its contents are understood as they deserve to be is hardly probable from what we know of the mental character of our pupils and young practitioners. It is a hard book to read and comprehend, albeit its style is at once chaste and classical. Principles are, of all things, the most difficult for a student to learn. He can easily comprehend a recipe, or a formula, for calomel and jalap, and he is sure to treasure up everything of this kind in his mind with a sort of miserly care, as if the loss of it would be ruinous to his future career; but if we attempt to teach such a youth a great principle, he looks at us with a holy horror, and forthwith regards us as his worst enemy. An impatience to get at what is practical, and to study only what is practical, is eminently characteristic of the medical mind of the United States at this time. Young Physic will not stop to think and reflect; it imposes upon itself no restraint, and will tolerate no advice; it must complete in a year what it took men a quarter of a century ago to complete in three or four times that period. Let a teacher attempt to instil principles in the class-room, and he will soon find himself without auditors. No lecturer was ever more unpopular, on this account, with first course students than the late Professor Drake. It was his constant practice to devote nearly one-third of his course to the discussion of the great principles of medicine, and we often heard him remark how impossible it was to engage the attention of the young men who attended his prelections, so few of them being educated up to that point. The second course students, on the other hand, having made greater proficiency in the several branches of medicine, generally listened to him with the greatest respect and interest. A similar difficulty seems to be felt in Great Britain, if we may judge from the following statement of the author: "When first," he says, "I communicated to my publisher my intention of bringing out a work on the Principles of Medicine, I was by no means encouraged by the intimation that books on that subject did not sell; but no sooner was it discovered that the 'Principles' were essentially and intelligibly *practical*, than the demand for the work became sufficient to exhaust two editions, long before I could find time to replace them."

We consider that Messrs. Blanchard & Lea have done the profession of this country a great service by furnishing them with a reprint of this admirable production, which has placed its author in the front rank of British pathologists. We trust that edition after edition will be exhausted, until, at no distant day, some American physician, charged with competent knowledge, and stimulated by a noble ambition, shall be able to supply us with a similar treatise at least equally meritorious.

